# Recent Advances in 3D Bioprinting of Porous Scaffolds for Tissue Engineering: A Narrative and Critical Review

**DOI:** 10.3390/jfb16090328

**Published:** 2025-09-04

**Authors:** David Picado-Tejero, Laura Mendoza-Cerezo, Jesús M. Rodríguez-Rego, Juan P. Carrasco-Amador, Alfonso C. Marcos-Romero

**Affiliations:** 1 Departamento de Expresión Gráfica, Escuela de Ingenierías Industriales, Universidad de Extremadura, Avenida de Elvas, s/n, 06006 Badajoz, Spain; davidpicateje@unex.es (D.P.-T.); lmencer@unex.es (L.M.-C.); jpcarrasco@unex.es (J.P.C.-A.); acmarcos@unex.es (A.C.M.-R.); 2 Departamento de Bioquímica, Facultad de Ciencias, Universidad de Extremadura, Avenida de Elvas, s/n, 06006 Badajoz, Spain

**Keywords:** tissue engineering, 3D-bioprinting, 3D design, porous scaffolds, bioink

## Abstract

3D bioprinting has emerged as a key tool in tissue engineering by facilitating the creation of customized scaffolds with properties tailored to specific needs. Among the design parameters, porosity stands out as a determining factor, as it directly influences critical mechanical and biological properties such as nutrient diffusion, cell adhesion and structural integrity. This review comprehensively analyses the state of the art in scaffold design, emphasizing how porosity-related parameters such as pore size, geometry, distribution and interconnectivity affect cellular behavior and mechanical performance. It also addresses advances in manufacturing methods, such as additive manufacturing and computer-aided design (CAD), which allow the development of scaffolds with hierarchical structures and controlled porosity. In addition, the use of computational modelling, in particular finite element analysis (FEA), as an essential predictive tool to optimize the design of scaffolds under physiological conditions is highlighted. This narrative review analyzed 112 core articles retrieved primarily from Scopus (2014–2025) to provide a comprehensive and up-to-date synthesis. Despite recent progress, significant challenges persist, including the lack of standardized methodologies for characterizing and comparing porosity parameters across different studies. This review identifies these gaps and suggests future research directions, such as the development of unified characterization and classification systems and the enhancement of nanoscale resolution in bioprinting technologies. By integrating structural design with biological functionality, this review underscores the transformative potential of porosity research applied to 3D bioprinting, positioning it as a key strategy to meet current clinical needs in tissue engineering.

## 1. Introduction

The extracellular matrix (ECM) is a dynamic network of proteins, carbohydrates and other molecules that regulates key cellular functions, including signaling, morphology and organization cellular in the tissue. Its tissue-specific composition not only provides structural support, but also influences critical biological processes such as tissue regeneration and repair [1,2]. These multifaceted attributes have inspired bioengineering, particularly in the field of tissue engineering, which seeks to replicate the characteristics of the ECM to overcome the limitations of conventional transplantation [3].

The development of bioactive materials, designed to interact favorably with the biological system, have proven to be essential for promoting the formation of new tissues [4]. Among them, hydrogels have gained particular relevance due to their highly hydrated three-dimensional structure, which exhibits physical and biological properties similar to those of the ECM [5,6,7]. Nevertheless, the development of customized scaffolds that effectively integrate appropriate biological and mechanical properties remains a critical challenge today [8].

In tissue engineering, a scaffold is a three-dimensional structure designed to support the growth and organization of new tissue. It acts as a temporary extracellular matrix, mimicking the mechanical and biological characteristics of natural tissue to promote integration and formation of new tissue [9]. Scaffolds must not only exhibit good biological qualities, such as biocompatibility, bioactivity, biodegradability or the ability to promote cell invasion, adhesion and proliferation; they must also exhibit tissue-specific mechanical qualities such as tensile strength, stiffness, elastic modulus, viscoelasticity and porosity [5,10].

Porosity is a crucial factor in scaffold design, significantly influencing not only the mechanical and biological properties of the material, but also the scaffold’s physio-thermal properties and internal transport dynamics. Key pore characteristics (such as size, shape-geometry, spatial distribution, and interconnectivity) play a vital role in determining cellular behavior. These characteristics regulate the diffusion of nutrients and oxygen, as well as the removal of metabolic waste, both of which are essential for maintaining cellular viability throughout the entire scaffold [11,12].

Moreover, pore size and geometry affect cell adhesion, as specific dimensions and surface curvatures can enhance or inhibit focal adhesion formation depending on the cell type. The distribution and interconnectivity of pores directly influence cell migration and proliferation, enabling cells to infiltrate and colonize the entire scaffold volume [13]. This migration is critical not only for homogeneous tissue formation but also for the development of organized structures such as blood vessels (angiogenesis), bone canaliculi, and neural networks. Additionally, certain pore configurations can modulate cellular alignment, polarization, and differentiation by providing mechanical cues and spatial constraints that mimic native extracellular matrix (ECM) environments [10,11,12,13,14]. Also, porosity is highly connected to the scaffold comfort property, as the parameter affects moisture and breathability, mechanical softness and flexibility, thermal regulation, and tissue–patient interaction [15,16,17,18].

Therefore, understanding and precisely tuning porosity parameters is essential for guiding specific cellular responses (such as osteogenic, chondrogenic, or neurogenic differentiation) [19], depending on the intended application of the scaffold.

Consequently, mastering porosity design strategies is crucial for the development of advanced scaffolds tailored to specific tissue engineering applications, enabling the creation of more physiologically relevant constructs for personalized regenerative therapies.

**Figure 1:**
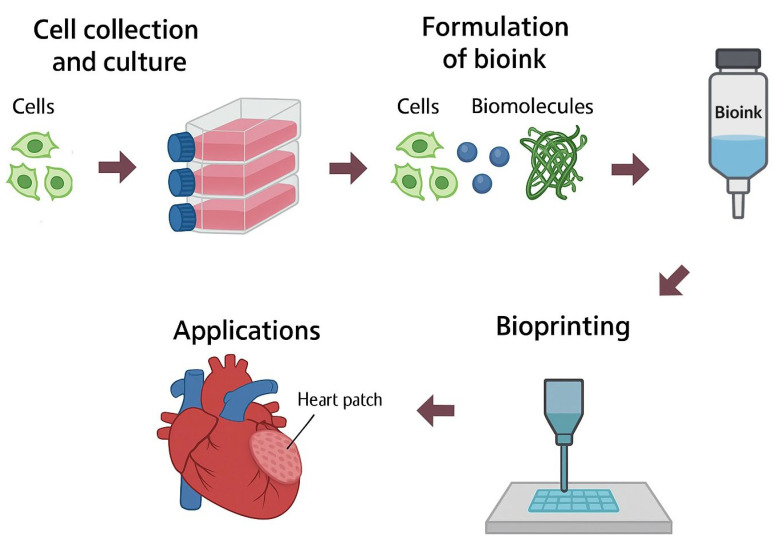
Schematic representation of 3D bioprinting, describing the process of cell culture and bioink formulation.

The advent of advanced technologies such as 3D bioprinting has revolutionized scaffold design by enabling precise control over porosity parameters. Three-dimensional bioprinting is defined as a technique for fabricating biomimetic structures using cell-laden biomaterials deposited in predefined patterns through a layer-by-layer process [20] (Figure 1). This technology allows for the customization of geometries and porosity gradients via computer-aided design (CAD), thereby optimizing both the mechanical and biological performance of the scaffold [21,22,23].

While previous reviews have typically addressed scaffold porosity by examining isolated factors, such as pore size or a single bioprinting technique, the present work adopts a comprehensive perspective. It analyses the main porosity-related parameters (size, interconnectivity, geometry, and distribution) and discusses their combined influence on 3D bioprinting outcomes and scaffold biocompatibility. Furthermore, the review highlights recent advances, points out methodological limitations, and proposes an evaluation framework aimed at enabling more consistent cross-study comparisons and guiding the design of functional scaffolds.

Within this context, the objective of the review is to provide a narrative and critical synthesis of the latest progress in the design and fabrication of porous scaffolds, with a particular emphasis on the role of porosity in shaping both biological and mechanical performance. To achieve this, 112 articles were systematically analyzed to identify current challenges, highlight emerging trends, and propose future research directions.

To ensure an up-to-date and coherent overview, publications were primarily retrieved from the Scopus database covering the period from 2014 to June 2025, using the keywords “porous scaffold” and “3D bioprinting.” Searches were refined with related terms such as “tissue engineering” and “3D printing.” Articles were selected based on their relevance, novelty, and data quality, resulting in 112 works forming the core body of analysis. Additional references were incorporated during peer review to enrich the discussion and reflect recent advances.

## 2. Porosity

The International Union of Pure and Applied Chemistry (IUPAC) defines porosity as “a concept related to texture, referring to the pore space in a material” [24]. In the field of Materials Science, and in order to avoid ambiguities and to properly contextualize the present work, it is essential to distinguish between two types of porosity: on the one hand, intrinsic porosity, which arises from the chemical composition and molecular structure of the material; and on the other hand, induced porosity, which results from the manufacturing or processing techniques to which the material has been subjected.

### 2.1. Chemically Derived Porosity or Molecular Porosity

Chemically derived porosity is determined by the molecular structure and internal organization of the material. A given material may exhibit nanometric-scale porosity as a consequence of its crystalline or amorphous organization, its degree of molecular packing, or—specifically in the case of hydrogels—the level of crosslinking and the concentration of chemical components [25]. All these factors affect the material’s texture and define the presence of voids at the submicrometric scale [26].

From a structural perspective, the porosity of a material can be classified into two main categories: intraparticle and interparticle porosity, depending on its location and morphological origin.

- Intraparticle porosity refers to the voids confined within a particle, microstructure, or polymer network. These pores may be closed, open, or interconnected, and their size can fall within the micro or nanometric range depending on the synthesis method and the base material [27]. This type of porosity is essential in adsorbent materials such as activated carbon, widely used in purification and filtering processes for gaseous or liquid contaminants, as in the adsorption of toxic compounds present in tobacco smoke [28]. At the biological level, although the term porosity is not strictly applied, internal functional cavities have been described in transmembrane proteins such as the Na^+^/K^+^-ATPase, whose conformational structure enables the opening and closing of ion channels, allowing for the active transport of ions against the electrochemical gradient [29].
- Interparticle porosity, in contrast, corresponds to the void spaces generated between particles, fibers, or polymer chains. This type of porosity depends mainly on the size, morphology, and packing density of the structural units, as well as the interaction forces between them [30]. In molecular or colloidal systems, these interactions determine the degree of compaction and therefore the volumetric fraction of free pore space [31]. A representative case is found in cementitious materials, where hydration leads to the formation of new solid products that generate an interconnected porous network, modifying the mechanical properties and permeability of the concrete [32]. Another example can be seen in hydrogels, which are materials composed of three-dimensional networks of hydrated polymers, where interparticle porosity is regulated by polymer concentration, degree of crosslinking, and the drying methods used. Techniques such as freeze-drying or solvent drying remove water and generate secondary pores, which are useful for tuning the structure and functionality of the material [33].

The porosity derived from internal structural organization is a critical parameter in biomaterial design, as it determines key properties such as solute diffusion, cell adhesion and migration, drug retention, and gas exchange [30]. In tissue engineering applications, particularly, pores in the micrometric range are of greater biological relevance, as they facilitate essential processes such as cell infiltration, nutrient transport, and extracellular matrix formation. Nanoporosity, on the other hand, although it also influences cell interaction and biomolecule absorption, cannot be precisely controlled by current additive manufacturing techniques, whose resolution lies primarily in the micrometric range. In this regard, recent trends have achieved nanometric-scale polymeric structures, using the electrospinning technique [34,35].

Overall, the present work will focus on the analysis and control of porosity at micrometric scale, which is more directly related to biological functions and the customization potential of biofabricated structures, while acknowledging that many of the conclusions drawn may also be extrapolated to the intrinsic chemical porosity of various materials, given the functional interrelation between both types.

### 2.2. Processing-Induced Porosity

Processing-induced porosity, such as that generated through 3D printing techniques, refers to structural or macroscopic porosity, the scale of which depends directly on the manufacturing parameters, typically ranging from micrometers to millimeters [36,37].

In many instances, the use of the term “porosity” may lead to ambiguity if the specific type being addressed is not clearly stated, since it encompasses very different physicochemical realities, as many authors acknowledge [36,38,39].

Accordingly, we define Porosity as the presence of interconnected open spaces within a material [40,41]. Mathematically, porosity is expressed as the ratio of the density of the scaffold (*ρ_s_*) to the density of the base material (*ρ_b_*) (Equation (1)).

(1) $$R=100\times \left(1-\frac{\rho_{s}}{\rho_{b}}\right)$$

This feature is fundamental for bioengineered materials, as it directly influences their biological acceptability and cell integration. Aspects such as angiogenesis, calcification and cell degradation are highly dependent on the porosity of the material, which influences the topography of the structure [42]. This means that porosity affects the mechanical properties (stiffness, elastic modulus and compressive strength) and degradation rate of the scaffold, crucial elements for engineering applications tissue [12,42].

## 3. Pore Manufacturing

In porous scaffold fabrication, a distinction can be made between conventional and modern methods. Alternatively, these methods can be more simply classified as Additive or Subtractive. In some Subtractive methods, a sacrificial substance called Porogen is used, and its removal creates the pores [43]. Conventional methods include Solvent Casting, Particle Leaching, Freeze-Drying, Thermal-Induced Phase Separation (TIPS), Gas Foaming and Electrospinning. Except for electrospinning, these conventional methods offer limited control over the chemistry, internal structure, and design of complex architectures [37]. Additionally, some methods may leave behind remnants of cytotoxic compounds [42]. None of these methods -not even Electrospinning- can generate controlled and precise pore interconnection and simultaneously offer good mechanical properties [44,45]. For example, the foam replication method can achieve high levels of pore interconnectivity, but it cannot be tailored to individual patients, and often defects introduced during the process result in poor mechanical properties [46].

Modern methods, also known as rapid prototyping, include according to the American Society for Testing and Materials (ASTM) 7 different types of processes: Photopolymer vat, Material Jetting, Material Extrusion, Powder Bed fusion, Direct Energy Deposition, Sheet lamination and Binder Jetting [21]. Additive manufacturing methods offer greater precision by utilizing Computer-Aided Design (CAD) modeling, enabling control over the internal structure at both macro and microscopic levels. This allows for the creation of 3D scaffold models tailored to specific patients [47] and enables their mechanical properties to be optimized [22,23].

In this context, 3D bioprinting has emerged as a revolutionary technology in tissue engineering. This approach makes possible the precise fabrication of scaffolds using computer-aided modeling (CAD) and hydrogel-based polymeric bioinks [7]. Using clinical images, such as magnetic resonance imaging (MRI) or micro-computed tomography (micro-CT), three-dimensional structures can be designed to closely mimic patient-specific tissue characteristics [9]. In addition, this technology offers the potential to integrate vascular systems into tissues, a key advance in the fabrication of functional organs and applications in orthopedics and dental implants [48].

Finally, it is important to note that each material possesses its own intrinsic porosity, as observed in hydroxyapatite scaffolds, where the granule size can partially influence the overall porosity [49,50]. Similarly, in hydrocolloid systems, the internal pore size is directly affected by solute concentration, with higher concentrations typically resulting in larger pore sizes [51,52]. In hydrogels, solute concentration becomes a critical factor in defining the internal architecture, enabling pore sizes to range from the nanometer to the micrometer scale. Other factors, such as the manufacturing method, also play a significant role in the development of scaffold porosity [53]. Given the challenges in porous scaffold fabrication and the difficulty of drawing general conclusions when materials inherently differ in their intrinsic properties, the present study will focus on those aspects of porosity that can be directly modified through Computer-Aided Design (CAD).

## 4. Pore Characteristics

### 4.1. Pore Size

Pore size is a critical variable for scaffold properties, directly influencing key biological processes such as cell adhesion, migration, and angiogenesis. The IUPAC classifies pores based on physicochemical principles of adsorption: micropores (<2 × 10^−3^ µm), mesopores (2–50 × 10^−3^ µm), and macropores (>50 × 10^−3^ µm) [54]. Alternatively, the *Bureau International des Poids et Mesures* (BIPM) establishes a classification of high interest for nanotechnology, in which pores are divided into nanopores (0.1–100 × 10^−3^ µm), micropores (0.1–100 µm) and millipores (0.1–100 × 10^3^ µm) [39]. This approach is more suitable for tissue engineering, as it allows for the wide variety of scales required to interact with biological structures (Table 1), from macromolecules (nanometers) and cell types to supracellular structures such as blood vessels (milimeters).

**Table 1:** Comparison of the typical sizes of various molecules, cells, and cellular structures, providing a reference to understand the scale differences between these biological components. It should be noted that discrepancies between cellular organelles and supracellular structures can occur, reflecting the unique specializations of each cell type.

Type	Name	Aproximate Size (Diameter, µm)	Reference
Macromolecules	Albumin	0.007 µm	Tojo and Kinugasa (2012) [55]
	Cholesterol	0.0239 µm	PDBe-KB [56]
	Triglyceride	0.0015–0.002 µm (up to 1000 µm forming networks in Adipocytes)	A. Penagos et al. (2024) [57], Patterson (2009) [58]
	Antibodies	0.0035 µm	Meyer-Tamaki (2013) [59]
	Collagen molecule	0.0015 µm	Van den Berg (2012) [60]
	DNA chromatin fiber	0.005–0.023 µm	Ou et al. (2017) [61]
Cellular structures	Nucleus	10 µm	Sun et al. (2000) [62]
	Mitochondria (80S)	0.5–10 µm	Duranova et al. (2020) [63]
	Ribosome	0.025–0.030 µm	Khatter et al. (2015) [64]
	Vesicles	0.020–0.560 µm	Chernyshev et al. (2015) [65]
	Exosome	0.040–0.1 µm	Chen et al. (2019) [66]
Cellular types	Osteoblast	10–15 µm	Kassem et al. (1992) [67]
	Chondrocyte	12–18 µm	C. A. Pole (2019); Bush and Hall (2003) [68,69]
	Pancreatic ß-cell	35 µm	Ginzberg (2015) [70]
	Hepatocyte	40 µm	
	Keratocyte	45 µm	
	Fibroblast	85 µm	
	Adipocyte	110 µm	
	Erythrocyte	7–8 µm	Fabry (1981); Vömel (1980) [71,72]
	Lymphocyte	6–8 µm	Bagge and Braide (1982); Kuse et al. (1985) [73,74]
	Macrophage	21 µm	Krombach (1997); M. Naito (2008) [75,76]
	Monocyte	9–19 µm	Wang et al. (1992) [77]
Supracellular structures	Osteocyte canaliculi	0.2–0.42 µm	Kufahl and Saha; Rath Bonivtch et al. (2007) (1990) [78,79]
	Osteocyte lacuna	5–6 µm	Rath Bonivtch et al. (2007) [79]
	Aorta	25,000 µm	William C. Parke (2020); Connor et al. (2022); Tajeddin and Mustafaoglu (2021) [80,81,82].
	Artery	4000 µm	
	Arteriole	10–100 µm	
	Terminal arteriole	5–10 µm	
	Capillary	5 µm	
	Venule	10–200 µm	
	Vein	5000 µm	
	Vena cava	30,000 µm	

While pore classifications have been proposed in the context of tissue engineering (Table 2), a unified framework has yet to be established, as definitions vary depending on the experimental approach, the cell types studied, and the target tissue.

**Table 2:** The table presents a comparative analysis of pore size classifications according to various sources, providing different categories based on pore size (diameter).

Ranking	IUPAC [54]	Schwarz and Epple (1998) [83]	R.A. Perez and G. Mestres (2016) [84]	P. Habibovic et al. (2005) [85]	F. Junior Maksoud et al. (2022) [14]	M. Ebrahimi (2021) [42]
Macropore	>50 × 10^−3^ µm	>1 × 10^5^ µm	>5 × 10^4^ µm	-	(1–5) × 10^5^ µm	>1 × 10^5^ µm
Mesopore	(2–50) × 10^−3^ µm	-	-	-	-	-
Micropore	<2 × 10^−3^ µm	<1 × 10^3^ µm	<5 × 10^4^ µm	<1 × 10^4^ µm	0.1 µm–1 × 10^5^ µm	(1– 100) × 10^2^ µm
Submicropore	-	-	-	-	-	0.1–100 µm
Nanopore	-	-	-	-	<0.1 µm	<0.1 µm

Although there is no definitive consensus within the scientific community regarding the ideal pore size for each specific biological process, it is widely accepted that optimal pore dimensions depend on multiple factors, including cell type, target tissue, scaffold material, and the intended biological function [86,87] (Table 3). Pores ranging from 100 to 500 µm, typically produced through conventional fabrication techniques and characterized by random arrangement, are generally interconnected and promote essential biological processes such as cell migration, adhesion, tissue ingrowth, and vascularization.Conversely, pores smaller than 100 µm have a more limited effect on these processes but play a crucial role in facilitating the transport of ions and macromolecules, such as adhesion and signaling molecules [14,88]. However, this range is too wide and not specific enough. In applications more specific, pores > 150 µm have been shown to promote vascularization and osteogenesis by increasing oxygen supply, while smaller pores favor processes such as chondrogenesis and osteochondral ossification under hypoxic conditions [10,88,89]. In addition, scaffolds with gradients of pore size, rather than uniform sizes pattern, showed better biological and mechanical properties suggesting a synergy between the different porosity scales [86].

Finally, Contreras-Raggio et al. [90] highlighted that, although pore diameter is relevant for biological properties, its impact on mechanical behavior depends on the height-to-diameter (H/D) ratio. This parameter influences properties such as elastic moduli (stiffness) and yield strain, both of which are crucial for comparing biomaterials with varying geometrical proportions. These observations indicate that studying advanced geometric characteristics could be essential for optimizing customized scaffolds in tissue engineering.

**Table 3:** Representative examples of tissue-engineered scaffolds with corresponding achievements, pore size ranges, porosity values, and materials. Data include applications in bone, dental, vascular, cartilage, dermal, and neural tissues, highlighting the role of pore architecture in functional outcomes (adapted from the cited references).

Tissue Engineered	Achivement	Pore Size	Porosity%	Material	Reference
Bone and Dental	Cortical bone regeneration	<212 μm	27–37%	Titanium	C. Torres-Sanchez et al. (2017) [89]
		1–2 μm	-	Demineralised bone matrix	D. Henrich et al. (2015) [91]
	Trabecular bone regeneration	300–500 μm	54–58%	Titanium	C. Torres-Sanchez et al. (2017) [89]
	Osteoconductivity, Osteogenesis and Angiogenesis	>150 μm	-	Bioceramic based scaffolds	H. Jodati et al. (2020) [86]
	Osteogenesis	350 μm	-	Bioceramic based scaffolds	M. A. Velasco et al. (2015) [10]
	Bone marrow regeneration	100–500 μm	60%	b-TCP	D. Henrich et al. (2015) [91]
	Bone formation	300–635 μm	20–60%	Titanium	W. Xu et al. (2022) [92]
	Proliferation and differentiation of dental pulp stem cells	251–425 μm	-	PLLA	C. M. Conde et al. (2015) [93]
		65 μm	-	Collagen	Q. Zhang et al. (2022) [94]
		200–300 μm	-	Calcium Polyphosphate	F. M. Wang et al. (2006) [95]
	Dental neovascularization and osteogenesis	750–900 μm	85%	Hydroxyapatite	P. Li et al. (2023) [96]
Vascular	Vascular smooth muscle cells	60–150 μm	-	PLLA	Y. Wang et al. (2014) [97]
	Microvascular epithelial cells	38–50 μm	-	PLLA	J. Zeltinger et al. (2001) [98]
Cartilage	Angiogenesis and chondrocyte proliferation	280–340 μm	87–94%	PLGA and cell-Free Fat extract	J. Ding et al. (2024) [99]
	Chondrogenesis	100 μm	-	Bioceramic based scaffold	M. A. Velasco et al. (2015) [10]
		150–250 μm	-	Collagen type I	Q. Zhang et al. (2014) [100]
		90–250 μm	-	Silk fibroin	K. S. Han et al. (2015) [101]
	Chondrocyte proliferation	245 μm	-	Agarose and Snail mucus	V. A. Ajisafe et al. (2024) [102]
		100–300 μm	93%	Human-Like Collagen and Bovine Serum Albumin	X. Song et al. (2017) [103]
		90–250 μm	-	Silk fibroin	K. S. Han et al. (2015) [101]
*Dermal*	Keratinocyte differentiation	100–150 μm	-	Fish gellatin and Hyaluronic acid	P. Chailom et al. (2025) [104]
	Skin wound repair	130 μm	-	Collagen, Hyaluronic acid and Gelatin	H. M. Wang et al. (2013) [105]
	Fibroblast adhesion and proliferation	100–200 μm	>85%	Silk fibroin and hair-derived keratin	N. Bhardwaj et al. (2015) [106]
Neural	Peripheral axon generation	75 μm	-	Hexamethylene diisocyanate, Poly(epsilon-caprolactone) and Dianhydro-D-sorbitol	I. Bružauskaitė et al. (2016) [107]
	Schawn cells	20–50 μm	-	Collagen type I	A. Bozkurt et al. (2009) [108]
	Direct growth of the neurons	20–30 μm	-	Collagen coated photosensitive polyimide.	M. J. Mahoney et al. (2005) [109]
	Neuronal differentiation	50–350 μm	-	Chitosan	X. Yi et al. (2011) [19]
		95 μm	-	Collagen and Gycosaminoglycans	A. Kourgiantaki et al. (2020) [110]

### 4.2. Porosity Percentage (%)

**Figure 2:**
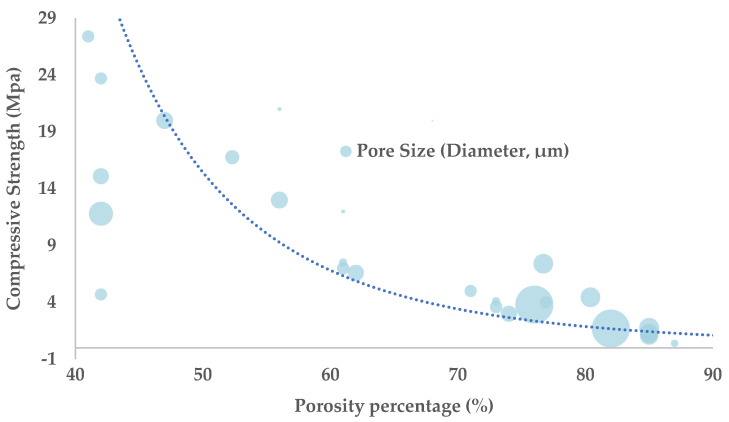
Evolution of Compressive Strength in relation to Pore Size (reference blue dot is equivalent to a 200 μm pore) and Porosity Percentage. As pore size increases, there is a noticeable decrease in compressive strength, indicating that larger pores weaken the structural integrity of the scaffold. Conversely, a higher porosity percentage also correlates with reduced compressive strength, as the increased void space within the scaffold compromises its ability to withstand compressive forces. Data source from Ref. [86].

The porosity percentage (Porosity%) is a critical parameter that, along with pore size, significantly impacts the mechanical and biological properties of the scaffold (Figure 2). Several studies agree that an increase in Porosity% reduces the mechanical properties of the scaffold, as higher void volume results in lower compressive strength, stiffness (Young’s modulus), yield strain and yield strength [23,84,90,111]. However, these observations cannot be generalized to all scaffolds, as other factors, such as the H/D ratio, the material, the scaffold design and the manufacturing process, also play a crucial role in the final material properties.

The decrease in stiffness and compressive strength caused by increased Porosity% has prompted the design of scaffolds capable of emulating bone load bearing, with optimal porosity values between 15–45% [112,113]. Scaffolds with a Porosity% above 40% exhibit mechanical properties similar to trabecular bone, whereas lower porosity values result in mechanical behavior more akin to cortical bone [86]. Furthermore, the compressive behavior of scaffolds with high porosity can be partially countered by optimizing the geometry and distribution of the pores along the material, adjusting them to the main stress directions [90,112,114]. From a biological perspective, a PCL scaffold with 50% porosity, 500 μm pores, and geometries such as square or gyroid closely mimics the characteristics of cortical bone [23]. However, generalizing conclusions remains challenging, as the optimal mechanical and biological properties can be achieved through various parameter combinations, depending on the scaffold design and specific conditions.

In vitro tests indicate that low Porosity% promotes cell aggregation and restricts proliferation, which can enhance early osteogenesis. Conversely, high porosity improves nutrient transport and cell expansion, leading to enhanced overall biological performance [88]. A study on polylactic-co-glycolic acid (PLGA) scaffolds incorporating 20% *w*/*w* β-tricalcium phosphate (β-TCP) and featuring gradient macroscopic channels—designed to mimic the periosteum and endosteum—revealed that porosities below 75% restricted tissue ingrowth, whereas porosities approaching 90% significantly enhanced it [115].

For any given value of Porosity%, the microstructure of the scaffold will determine the differences in stiffness and permeability. The effective permeability of a scaffold is strongly influenced by pore size and arrangement, highlighting the importance of applying advanced techniques such as 3D bioprinting and CAD modeling. These methods enable the precise optimization of microstructures, ensuring a balance between permeability and mechanical properties [116].

Ideally, scaffold design should achieve a harmonized balance between material degradation and tissue regeneration, ensuring that the degradation rate aligns with the natural regeneration process of the targeted in vivo tissue [84]. Pore size and Porosity% play a crucial role in determining the surface area available for cell-scaffold interaction, directly affecting the scaffold’s degradation rate. For highly biodegradable biomaterials, excessive porosity is not recommended, as it may compromise scaffold integrity before tissue regeneration is complete. In contrast, biomaterials with a low degradation rate and superior mechanical properties can benefit from higher porosity, enhancing host-tissue interaction and accelerating cell-mediated degradation [88].

### 4.3. Porosity Connectivity

**Figure 3:**
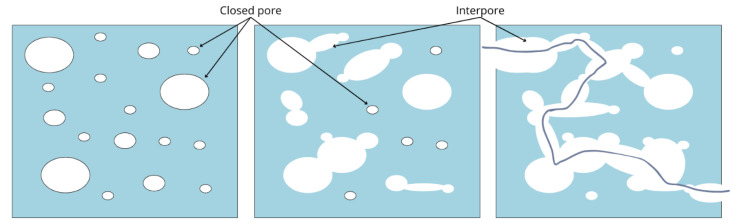
Schematic representation of three different scaffold designs based on pore interconnectivity. The first scaffold features fully closed pores, the second scaffold exhibits a combination of closed pores and partial inter-pore connections, and the third scaffold, which displays a high level of pore interconnectivity, also includes a blue line representing a hypothetical flow pathway within the scaffold.

As previously noted, pore size has a greater impact on biological properties than on scaffold mechanics. Likewise, pore interconnection plays a crucial role in determining how cells interact with their environment [86]. Interconnection refers to the connecting passage between two pores [84]. For clarity, this paper will refer to these connections as ‘Interpore’, although some authors use the term ‘throat connection’ [47]. This distinction is important, as narrow interpore sizes can create bottleneck effects, restricting cell mobility and potentially hindering tissue regeneration (Figure 3).

As a general rule, greater interconnectivity in scaffolds tends to reduce mechanical properties while enhancing biodegradation, cell migration, and proliferation [82]. In a study on cellular behavior at different interpore sizes, researchers analyzed osteoblast activity in hydroxyapatite and β-tricalcium phosphate ceramic scaffolds with 50% porosity, using interpore sizes ranging from 30 to 100 μm. Results showed that interpore sizes above 20 μm enabled cell penetration and chondroid tissue formation, with 40 μm being the optimal size for these processes. Additionally, at 50 μm and above, the formation of mineralized bone matrix was observed [117]. These findings highlight the crucial role of interpore density in scaffold performance. Another study examining the effect of pore interconnectivity on chondrocyte proliferation in chitosan and polyglycolic-acid based scaffolds (with pore sizes between 10 and 120 μm) concluded that larger interpore sizes improved metabolic activity and proliferation. This effect was attributed to enhanced nutrient diffusion and waste removal [118].

In the context of pore interconnectivity, two key concepts stand out: Percolation Diameter and Tortuosity. Percolation Diameter refers to the largest object that can move freely and indefinitely through a scaffold without obstruction [119]. Interpore sizes below 40 μm generally exhibit low cell motility, though this threshold varies depending on the specific percolation diameter of each cell type [119].

Tortuosity describes how the sinuosity and interconnectivity of pores influence the flow of liquids and gases within the scaffold [120]. The tortuosity index is one of the most widely used metrics for characterizing tortuosity in porous materials. It is defined as the ratio between the actual length of a pore pathway (or vessel segment) and the straight-line distance between its starting and ending points. Several methods exist to define tortuosity and relate it to the restricted diffusion coefficient in porous media, depending on the specific morphology and porosity under investigation [121].

Mathematically, tortuosity (*τ*) is expressed as Equation (2):

(2) $$\tau =\frac{L_{p}}{L_{s}}$$

where *L_p_* is the effective (actual) pore length and *L_s_* refers to the sample length.

Understanding and quantifying tortuosity is essential for controlling fluid transport within scaffolds. A higher tortuosity value implies more complex and winding pathways, which impede molecular flow, whereas lower tortuosity corresponds to more direct routes. Interestingly, increased tortuosity has been associated with enhanced cell adhesion and tissue regeneration, as it provides greater surface area for cell-material interactions. It also significantly influences scaffold permeability and the shear stresses experienced by resident cells [122].

Tortuosity also plays a direct role in determining the effective diffusion coefficient (*D_e_*), which quantifies the ability of molecules to diffuse through a porous matrix. It can be calculated using the following Equation (3) [123]:

(3) $$D_{e}=\frac{D_{B}\cdot \varepsilon \cdot f}{\tau }$$

where

- *D_B_* is the bulk diffusion coefficient, which depends on the molecular species and the solvent, but is independent of the scaffold’s structure;
- ε is the porosity of the scaffold;
- *f* is the diffusion–constriction factor; accounting for pore shape and connectivity;
- *τ* is the tortuosity index.

Accurate prediction of the effective diffusion coefficient requires a comprehensive understanding of scaffold tortuosity, as it governs molecular transport dynamics. These concepts are critical for interpreting key biological processes such as cell migration, scaffold colonization, and nutrient diffusion, especially in fields like bioengineering and drug delivery.

In this context, pore connectivity emerges as a critical determinant of net permeability, facilitating efficient fluid and solute flow throughout the scaffold. This, in turn, directly influences cell movement, adhesion, and proliferation. However, while increased pore connectivity enhances biological performance, it often compromises mechanical integrity by reducing stiffness and compressive strength. The magnitude of this trade-off depends on the combination of porosity parameters, intrinsic material properties, and scaffold design. Nevertheless, the mechanical impact of pore connectivity is generally less pronounced than that of total porosity percentage.

In conclusion, while pore interconnection plays a fundamental role in scaffold permeability and supports cell migration (particularly for interconnection diameters above 40 μm [117,119], current knowledge remains largely based on in silico models. Therefore, experimental validation is crucial for fully optimizing scaffold designs for biological applications.

### 4.4. Pore Shape (Geometry)

**Figure 4:**
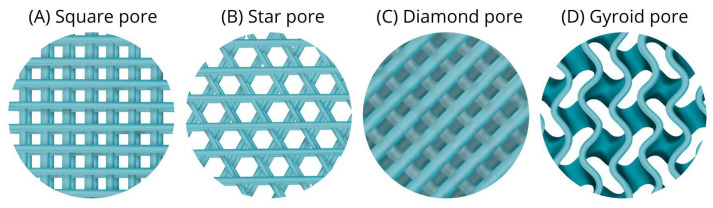
Schematic representation of four different pore geometries used in scaffold design. (**A**) Square pores, characterized by their uniform and angular structure, (**B**) Star-shaped pores, featuring a central point with radiating arms, (**C**) Diamond-shaped pores, with their elongated and angular form, (**D**) Gyroid pores, exhibiting a complex, continuous, and triply periodic minimal surface.

The mechanical properties of a scaffold must closely match those of the target tissue it aims to regenerate. Conventional scaffold manufacturing techniques lack the ability to control pore distribution and geometry [37], highlighting the importance of additive manufacturing methods that incorporate computer-aided design (CAD) processes. These approaches allow the creation of pores with diverse geometries (Figure 4)—such as spherical, cubic, prismatic, pyramidal, star-shaped, X-pattern, diamond, or gyroid—which impart specific mechanical and biological characteristics to the scaffold.

On the mechanical side, scaffolds with high porosity generally exhibit reduced compressive strength. However, certain geometries, such as the gyroid structure, help retain mechanical performance even at high porosity levels [112]. Scaffolds with square-shaped pores can resist higher forces in the elastic region with less deformation compared to star-shaped or gyroid pores, regardless of the percentage of porosity studied. This indicates that square pores are stiffer than the other shapes, which are expected to exhibit lower tensile forces and lower elastic elongations. Therefore, square pores are recommended for tissues that withstand tensile forces, such as hard cartilage or bone [23]. Conversely, star-shaped pores tend to have lower interconnectivity, which can lead to reduced cell viability in culture [112].

At lower porosity levels (above 15%), scaffolds with diamond and gyroid pore geometries demonstrate superior compressive behavior compared to other designs. Both geometries exhibit higher compressive strengths and moduli than scaffolds with square or spherical pores. Notably, within the 15–30% porosity range, diamond-shaped pores achieve the highest compressive modulus. At porosity levels above 45%, diamond and gyroid structures show comparable mechanical properties [112].

Regarding biological performance, Khajehmohammadi et al. [23] reported high cell viability with 500 μm pore sizes and 50% porosity in gyroid and square pore geometries. These findings suggest that this combination may offer a promising balance between cell viability and mechanical properties, making it a potentially valuable approach for polycaprolactone (PCL) bone scaffolds [23]. Moreover, the surface roughness of gyroid and diamond geometries is higher than that of other pore structures, which significantly enhances cell adhesion and proliferation [112]. The diamond-shaped geometry demonstrated higher Bone Marrow Mononuclear Cell (BMSC) activity compared to star, normal, and gyroid shapes. Additionally, it exhibited greater compressive modulus and strength than the other tested geometries [112] and was observed to accelerate bone formation [122,124].

Another crucial factor is biodegradation. As with porosity percentage, matching the degradation rate of the scaffold to that of native tissue is vital to ensure that the scaffold resorbs at a pace compatible with tissue regeneration [125]. Since biodegradation is primarily influenced by the surface contact area between cells and the scaffold, pore geometry plays a major role. For instance, scaffolds with star or square geometries tend to degrade faster than those with gyroid structures [23].

Pore geometry has thus garnered significant attention for its profound influence on both the mechanical and biological properties of scaffolds. Among the various architectures studied, diamond and gyroid geometries stand out as the most effective in maintaining superior compressive behavior and strength compared to square and spherical geometries [112]. This makes them ideal candidates for scaffolds intended for load-bearing applications.

Finally, intentional micropore design not only optimizes internal transport of cells and nutrients but also helps mitigate adverse effects associated with other porosity parameters, such as pore size or overall porosity percentage. Thoughtful control of pore geometry contributes to a more balanced and efficient scaffold performance, both mechanically and biologically.

### 4.5. Pore Distribution

Using traditional scaffold manufacturing methods, pore distribution tends to be random, resulting in poorer mechanical properties compared to scaffolds with identical porosity but controlled distribution [126]. The advent of computer-aided additive manufacturing (AM) processes has introduced the ability to finely control pore distribution [84], leading to significant improvements in scaffold performance.

Advanced design strategies, such as Triply Periodic Minimal Surfaces (TPMS) and Voronoi tessellation, now enable the fabrication of scaffolds with precisely defined porosity, distribution, interconnectivity, and pore geometry [113,127]. Each method offers distinct advantages and challenges (Table 4). TPMS are three-dimensional, mathematically defined surfaces that repeat periodically in space and minimize local surface area without self-intersections [128]. Due to their high surface-to-volume ratio, they promote enhanced pore interconnectivity and fluid diffusion, making them highly attractive for scaffold design [129,130]. In contrast, Voronoi-Tessellation is based on the division of space into polygonal regions to give rise to complex structures similar to those found in biological samples allowing the creation of elements more biomimetic compared to TPMS, by better representing the heterogeneity of pore distribution in tissues [113,131,132].

**Figure 5:**
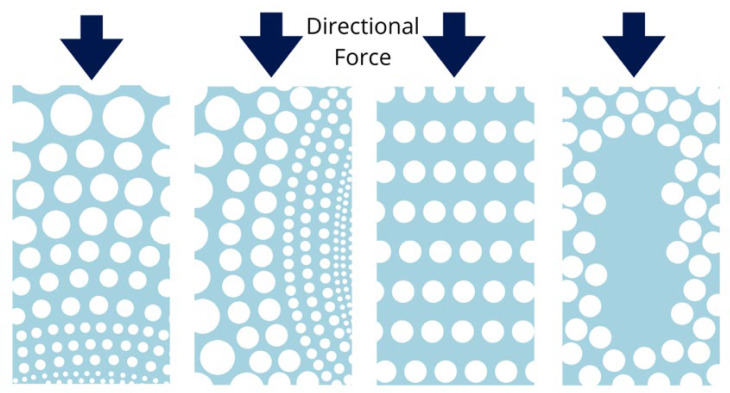
Schematic representation of gradient pore distributions with varying size and porosity. From left to right: radial gradient (perpendicular to the force direction), longitudinal radial gradient, vertically aligned pores, and high-density core.

Moreover, variations in pore distribution patterns significantly impact deformation mechanisms (Figure 5). Scaffolds with radial gradients oriented perpendicular to the loading direction exhibit improved deformability, while those with longitudinal radial gradients aligned with the loading axis tend to fail under lower stress levels, displaying brittle behavior [133]. Vertically aligned pores, arranged perpendicular to the stress direction, offer good load-bearing capacity but are more prone to stress concentration [126]. Additionally, scaffolds with denser cores and more porous surfaces show enhanced mechanical properties, particularly in terms of compressive strength, compared to those with homogeneous porosity distributions [134]. However, increasing core density may restrict nutrient diffusion and compromise cell viability, highlighting the trade-off between mechanical strength and biological functionality.

Thus, pore distribution is a critical design parameter, primarily influencing the scaffold’s mechanical behavior rather than its biological response. Generally, radial porosity gradients (perpendicular to the stress direction) favor greater deformability, whereas longitudinal gradients (parallel to stress) lead to earlier mechanical failure [126]. Structurally, aligning pores perpendicular to the stress direction maximizes load-bearing capacity.

An effective strategy to enhance mechanical performance is to reduce core porosity; however, this must be carefully balanced against the need for adequate nutrient transport to maintain cell viability. Ultimately, fine-tuning pore arrangement, size, and quantity enables the design of scaffolds with higher functional and regenerative complexity. For example, Tien et al. [135] successfully developed a multilayer gradient chitosan fiber scaffold capable of regenerating skin tissue by precisely controlling pore size, porosity levels, and spatial distribution, resulting in both enhanced mechanical strength and improved cellular infiltration.

## 5. Porosity Assessment Techniques

A wide range of equipment and software tools is available to assess the porosity and pore size distribution of softer scaffolds in the field of regenerative medicine [136]. Fluid intrusion methods, such as mercury intrusion porosimetry, liquid displacement, and capillary flow porometry, are commonly used for indirect porosity measurements, each offering different levels of sensitivity depending on pore size and scaffold material.

In addition to these physical techniques, imaging-based approaches like scanning electron microscopy (SEM) and microcomputed tomography (micro-CT) provide detailed, spatially resolved visualizations of the scaffold’s internal architecture, enabling quantitative analysis of pore morphology, distribution, and interconnectivity. These complementary methods together allow for a comprehensive understanding of scaffold porosity, which is essential for optimizing performance in tissue engineering applications.

### 5.1. Mercury Porosimetry

Mercury intrusion porosimetry enables the determination of the void fraction (total pore volume fraction), the average pore diameter, and the pore size distribution. In this method, the scaffold is placed in a penetrometer and infused with mercury under progressively increasing pressures (up to 414 MPa), forcing the mercury to penetrate into the pores.

Smaller pores require higher pressures to be completely filled due to greater surface tension forces [137].

Although precise, this technique requires careful manual handling, especially for delicate scaffolds like hydrogels, where lower pressures must be used to avoid structural damage [138,139,140]. Furthermore, thin-section materials are often destroyed during the process, and the toxicity and cost of mercury must be carefully considered [141,142].

### 5.2. Liquid Displacement Method

This method involves immersing a porous scaffold into a container filled with a known volume of non-reactive liquid, commonly ethanol. As the scaffold is submerged, the liquid penetrates its pores, and the volume of displaced liquid is measured, allowing indirect calculation of porosity.

Although relatively simple and low-cost, this method provides valuable information on open porosity, especially in materials where direct imaging may not be feasible. However, it is crucial to ensure complete liquid infiltration without altering the scaffold’s structure [143,144,145].

### 5.3. Capillary Flow Porometry

Capillary flow porometry is a non-destructive technique used to measure pore size distribution and permeability by passing a non-reactive gas through the scaffold under controlled pressure. It measures flow rate and pressure drop to infer pore sizes and can be performed under dry or wet conditions.This method is ideal for fragile or nanostructured materials, as it operates at low pressures and provides insights into pore size, connectivity, and tortuosity [146].

### 5.4. Scanning Electron Microscopy (SEM) Analysis

SEM provides high-resolution (nanometer-scale) images generated by scanning the scaffold’s surface with an electron beam after applying a conductive coating. Software tools like ImageJ (v1.52k 29) allow for rapid quantification of porosity based on cross-sectional images [147].

The advent of advanced image-processing algorithms has further enhanced SEM analysis by reducing manual intervention, saving time, and minimizing human error [148,149,150]. Moreover, the integration of artificial intelligence (AI) holds promise for future improvements through automated segmentation and feature recognition [151]. However, SEM is less suitable for sensitive, hydrated, or brittle materials due to sample preparation requirements (freezing and metal coating) that may introduce artifacts and alter the native microstructure [152]. Nonetheless, SEM remains widely considered the “gold standard” for porosity analysis in many contexts [153].

### 5.5. Microcomputed Tomography (CT) Imaging

Micro-CT imaging is a non-destructive, three-dimensional technique capable of accurately visualizing the internal architecture of porous scaffolds by detecting X-ray attenuation variations.

It enables quantitative analysis of pore size, distribution, and interconnectivity without altering the sample [154]. With minimal preparation required, micro-CT is particularly valuable for delicate or hydrated biomaterials [155]. In some cases, contrast agents may be employed to improve visualization.

Micro-CT enables multiscale analysis, from millimeter resolution (clinical systems) to submicron detail (nano-CT), supporting computational modeling and the design of bioinspired scaffolds [154]. However, limitations such as low contrast in soft materials, imaging artifacts in dense structures, and significant data processing requirements must be considered. Although real-time monitoring is possible, portability remains limited. Nevertheless, ongoing advancements continue to improve resolution and expand laboratory accessibility.

## 6. Discussion

### 6.1. Critical Analysis of the State of the Art

Mimicking the extracellular matrix (ECM) remains a complex challenge that extends beyond replicating molecular composition or structural resemblance. It demands a holistic approach that accounts for the dynamic and reciprocal interactions between cells and the ECM. While traditional strategies prioritized biomaterial chemistry and scaffold morphology, more recent advances emphasize the critical roles of cell penetration, oxygen and nutrient diffusion, and waste removal in supporting tissue regeneration [1]. In this context, scaffold porosity has emerged as a central design parameter, representing a crucial point of convergence between mechanical performance and biological functionality. This evolution requires re-evaluating characteristics once considered merely “desirable” and recognizing them as essential to scaffold design.

Despite these advances, significant limitations persist that must be addressed to move the field of porous scaffolds for tissue engineering forward. First, there is a pressing need to establish common characterization criteria. Even basic classifications, such as pore size ranges (Table 2), are often based on arbitrary thresholds tailored by individual authors to their specific research needs. This lack of consensus inherently complicates comparisons across studies.

**Figure 6:**
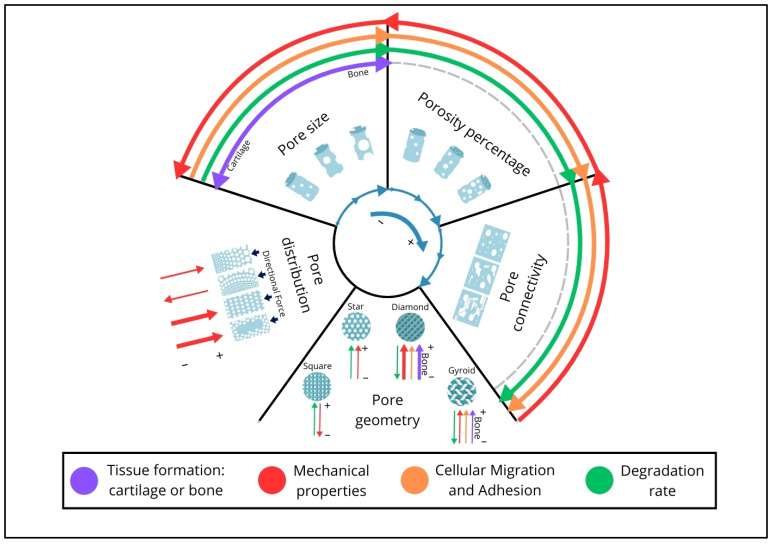
Schematic representation illustrating the relationships between the studied porosity parameters (Pore Size, Porosity Percentage, Pore Connectivity, Pore Geometry, and Pore Distribution) and their impact on biological aspects (Tissue Formation, Cellular Migration and Adhesion, and Degradation Rate) as well as general mechanical properties. The blue arrows in the inner circle indicate the increase in porosity parameters (Pore Size, Porosity Percentage, and Pore Connectivity), progressing in a clockwise direction. The arrows in the outer circles represent the behavior of the corresponding aspects according to their respective colors. In the case of Pore Distribution and Pore Geometry, as these are non-quantitative characteristics, both are arranged radially, with line thickness indicating the degree of observable effect and direction indicating whether it increases (pointing toward the center) or decreases (pointing outward from the circles).

Moreover, the field would benefit from defining a minimum set of scaffold characterization standards. Currently, most studies focus narrowly on porosity percentage and/or pore size, frequently overlooking other critical parameters emphasized in this work, including pore interconnectivity, distribution, and geometry (Figure 6). Even in biological assessments—where comparisons based on cytotoxicity and cell differentiation are relatively standardized—mechanical characterizations remain inconsistent. Some studies report only Young’s modulus, while others focus solely on compressive strength, making meaningful comparisons between scaffolds exceedingly difficult.

Understanding how different materials behave under comparable pore sizes and porosity percentages (and drawing consistent conclusions) thus remains a complex task. This difficulty is unsurprising given that mechanical performance at a given porosity level is strongly influenced by the intrinsic mechanical properties of the biomaterial itself [51] and the fabrication process used [53]. Consequently, detailed mechanical comparisons between scaffolds made from different materials, such as collagen, polylactic acid (PLA), or hydroxyapatite, are inherently challenging. Even within scaffolds fabricated from the same base material, significant differences in mechanical stability can be observed depending on formulation variables, such as collagen concentration (e.g., 1 wt% vs. 3 wt%) (Table 5).

This situation highlights the urgent need for standardized scaffold characterization protocols to enable more reliable and reproducible comparisons across studies. It also reinforces the critical role of fabrication methods in determining scaffold mechanical behavior [157,163], alongside key porosity design parameters such as pore geometry, size distribution, and interconnectivity.

Regarding the most recent advances, current efforts are focused on additive manufacturing technologies, particularly due to their compatibility with CAD systems. Top et al. [164] recently advocated for the use of PLA scaffolds with icosahedral geometry for bone regeneration, finding that they offered a better balance between porosity, mechanical properties, and dimensional accuracy compared to ABS-printed scaffolds. Meanwhile, Altunbek et al. [165], proposed a zigzag/spiral PCL cage design combined with poly (ethylene glycol) hydrogel loaded with human mesenchymal stem cells, resulting in a scaffold with enhanced mechanical strength and optimal nutrient diffusion. Their approach presents a promising personalized solution for treating critical-size bone defects. Increasingly, more complex and innovative scaffold designs are emerging, aiming to overcome the limitations of traditional implant materials and offering promising outcomes for tissue engineering.

These examples reinforce the critical role of porosity tuning in optimizing scaffold performance for regenerative medicine applications.

### 6.2. Use of Graphical Simulations in Design

The development of scaffolds for tissue engineering applications necessitates a precise design that ensures biomechanical and biological properties suitable for integration into the cellular environment. Computational simulations have emerged as essential tools in this context, enabling the prediction, optimization, and customization of these devices to meet the specific requirements of the tissue to be regenerated [166,167]. Computational modeling enables the evaluation of crucial factors such as mechanical strength, perfusion, and degradation behavior of materials. This approach provides valuable insights before experimental fabrication [168]. Through simulation, adjustments can be made to structural parameters such as porosity, interconnectivity and fiber orientation, which directly influence the cellular response and functionality of the scaffold.

One of the most employed techniques in scaffold simulation is Finite Element Method (FEM) analysis. This technique enables the evaluation of stress and strain distribution within the scaffold under various loading conditions, aiding in the identification of optimal designs that minimize structural collapse and maximize mechanical stability [169]. Khajehmohammadi et al. (2023) [169], highlight how FEM analysis applied to scaffolds made of poly-ε-caprolactone (PCL) allows accurate prediction of their mechanical properties, ensuring that the structures are able to withstand the physiological forces they will be subjected to in the human body. In addition, this approach allows the simulation of specific clinical scenarios, such as implantation in bone or cartilage regions, where the compressive strength and elasticity of the material play a crucial role.

In addition to structural analysis, computational simulations are used to assess fluid flow within the scaffold, a key determinant of cell viability and promotion of angiogenesis. Computational Fluid Dynamics (CFD) based models allow the distribution of nutrients and oxygen through the pores to be studied, ensuring that the design favors a suitable environment for cell proliferation [170].

The combined use of FEM and CFD analysis has allowed the development of optimization strategies in the fabrication of scaffolds, tailoring their morphological characteristics to specific physiological requirements. For example, recent studies have shown that variations in pore size and interconnectivity can significantly improve perfusion and thus the functionality of regenerated tissue [171].

The integration of artificial intelligence and machine learning in scaffold modeling represents a promising evolution in this field. Optimization algorithms can analyze large volumes of experimental and simulation data to generate highly customized and efficient designs. Furthermore, the use of 3D printing-based models combined with computational simulations allows the validation of theoretical results with physical structures, closing the loop between virtual design and real application [172].

### 6.3. Limitations

Drawing precise conclusions from the available literature remains challenging due to the absence of a standardized framework for accurately determining optimal pore sizes for specific cell lines, cell types, or processes. Similarly, there is no clear consensus on other fundamental parameters, such as porosity percentage, pore geometry, or pore distribution within the scaffold. Many studies focus exclusively on measuring pore size without considering interconnectivity or the overall impact of the microarchitecture, which limits the comparability of results across different investigations.

The lack of consensus on classifications proposed by various authors underscores the need for a unified classification system based not only on pore diameter but also on their biological function within the scaffold. This categorization should consider how each porosity configuration affects the mechanical properties of the material and cellular interaction, enabling a more rational design of scaffolds tailored to different biomedical applications.

Additionally, the intrinsic properties of the biomaterials used play a decisive role in the scaffold’s functionality. For instance, a scaffold made of collagen offers excellent biocompatibility but has inferior mechanical properties compared to a scaffold made of PLA, which, although structurally more robust, is less biodegradable. These inherent differences complicate the extrapolation of results and highlight the need for standardized characterization protocols to systematically evaluate each porosity parameter under controlled conditions.

To address this limitation, it would be essential to develop an experimental protocol for porosity characterization, in which each structural variable (pore size, porosity percentage, interconnectivity, etc.) is analyzed independently, keeping all other parameters constant within a predefined experimental structure. This approach would allow more precise correlations between the scaffold microarchitecture and its biological performance, thus facilitating the optimization of future design strategies in 3D bioprinting. In addition to bioengineering, advancements in porosity design would significantly benefit other fields where porosity plays a crucial role. These include biofilm formation [173], which has applications in wastewater treatment, nutrient diffusion, and filtering processes [174], as well as controlled drug release [175]. These principles are relevant for designing medical filters and are analogous to those analyzed for scaffold design in bioengineering applications.

In terms of manufacturing processes, 3D bioprinting has established itself as one of the most advanced techniques for obtaining porous scaffolds (tailored to a specific patient), thanks to its ability to precisely control the microstructure of the material through computer-aided design (CAD) [176,177]. However, this technology still faces significant limitations. Its implementation demands a high level of technical expertise, particularly in converting medical images (DICOMs) into functional 3D models. Additionally, while current bioprinting can achieve micron-level resolutions, it is not yet capable of producing structures with nanometer precision, which is crucial for applications in nanomedicine [178,179]. The improvement of deposition techniques and the optimization of systems photo-reticulation could help to overcome this technological barrier in the future.

The choice of manufacturing method significantly influences the micro- and nanoporous structural characteristics of a material. In the specific case of extrusion-based 3D bioprinting, the process generates shear stress that alters the arrangement and orientation of polymer chains, which can lead to a reduction in interparticle porosity due to compaction effects.

Fedorovich et al. [180], in their characterization of the 3D fiber deposition technique, observed that fiber spacing and deposition angle modulated both the overall scaffold porosity and its elastic modulus. On the other hand, studies on granular hydrogels—formulated from tightly packed microgels—have shown that this architecture facilitates extrusion but also undergoes internal reorganization during the printing process. This shear-induced rearrangement can result in transient stiffening of the material and a reduction in interparticle porosity, and in extreme cases, intraparticle porosity as well [181].

These findings highlight the currently limited understanding of the molecular-level impact of the bioprinting process, which hinders detailed analysis of cell–biomaterial interactions. A more precise characterization of these effects is essential to optimize the design of bioinks and bioprinted structures with functional properties suited for biomedical applications.

## 7. Conclusions

Porosity is a fundamental design parameter in scaffolds, directly affecting both biological and mechanical performance. Its relevance extends beyond tissue engineering to diverse applications such as biofilm formation for wastewater treatment, selective filtration in biomedical devices, and controlled drug delivery systems. These examples emphasize the need for unified characterization protocols to enable rigorous and cross-disciplinary comparisons.

Despite current challenges, 3D bioprinting represents a promising strategy to fabricate highly controlled porous architectures that closely mimic the microstructure of native tissues. Based on the analysis carried out in this review, the following key points are highlighted:

### 7.1. Current Limitations

- Lack of standardization in materials, pore geometries, cell types, and fabrication parameters.
- Scarcity of consistent data for tissues beyond bone and cartilage.

### 7.2. Required Actions

- Establish standardized methods for scaffold characterization (pore size, geometry, porosity percentage, interconnectivity, and spatial distribution).
- Develop tissue-specific porosity classifications considering cell interactions, adhesion molecule expression, and nutrient transport.
- Incorporate in silico simulations to predict scaffold performance under physiological conditions and optimize designs prior to fabrication.

### 7.3. Research Priorities

- Define robust correlations between scaffold microarchitecture and biological outcomes.
- Combine experimental studies with computational modeling, in vivo validation, multi-material bioprinting, and dynamic porosity approaches.
- Advance biomimetic 3D models that reproduce the mechanical and biochemical properties of native tissues for clinical translation.

Finally, future research should focus on printable and biocompatible biomaterials, such as advanced hydrogels, capable of achieving optimal biological and mechanical performance. Improving print fidelity and minimizing discrepancies between digital models and printed constructs remain critical priorities. The integration of computational simulations with clinical imaging (MRI, micro-CT) will be essential for the development of fully customized scaffolds adapted to patient-specific needs.

## Figures and Tables

**Table 4:** Features of TPMS (Triply Periodic Minimal Surface) and Voronoi-Tessellation scaffold designs.

	TPMS	Voronoi-Tessellation
Distribution of the units	Periodic and continuous repetition.	Random and discrete distribution.
Pore interconnection	Highly interconnected.	Heterogeneous, haphazard interconnection.
Mechanical resistance	High and highly controllable.	Depends on local seed distributions.
Applications	Bone scaffolds, filters, lightweight structures. Allows control of pore geometries, curvature levels and distribution.	Modeling of trabecular bone, soft tissues. Allows us to offer elaborated biomimetic micropatterns.

**Table 5:** Overview of how porosity parameters, such as pore size and porosity percentage, affect the mechanical performance of porous scaffolds fabricated from the same base material. It demonstrates that even small variations in internal architecture can lead to substantial differences in compressive strength and Young’s modulus. This highlights the critical role of porosity control in tailoring the mechanical properties of scaffolds to meet specific requirements, particularly in the context of tissue engineering and regenerative medicine.

Material	Pore Size (Diameter μm)	Porosity (%)	Young’s Modulus (GPa)	Compressive Strength (MPa)	Reference
Hydroxyapatite	300	42	-	4.7	I. Sabree et al. (2025) [156]
Hydroxyapatite	200–400	71	-	5	L. L. Wang et al. (2010) [157]
Hydroxyapatite (40 wt%) + PCL (60 wt%)	354	45	-	38.7	Y. S. Cho et al. (2019) [158]
PLA	200–1000	73	134.8	4.6	M. Alizadeh-Osgouei et al. (2021) [159]
PLA	1200–1300	73	108	2.7	
PLA	1000	40	-	51.3	H. Zhao et al. (2018) [160]
PLA	1000	70	-	5.1	
Collagen + bioglass	40–200	81	0.35	5.8	T. Long et al. (2015) [161]
Hyaluronan (70%) + Collagen (30%)	302.5	94%	7 × 10^−5^	-	A. Al-Munajjed et al. (2015) [162]
Hyaluronan (70%) + Collagen (30%)	402.5	94%	8.5 × 10^−5^	-	
Hyaluronan (70%) + Collagen (30%)	525	95%	9 × 10^−5^	-	
Collagen (0.25 wt%) + Chondroitin-6-sulphate (0.044 wt%)	58	98.9	3 × 10^−7^	-	C. M. Tierney et al. (2009) [52]
Collagen (0.5 wt%) + Chondroitin-6-sulphate (0.044 wt%)	92.95	99.3	5 × 10^−7^	-	
Collagen (1 wt%) + Chondroitin-6-sulphate (0.044 wt%)	111.02	98.8	9.4 × 10^−7^	-	

## Data Availability

No new data were created or analyzed in this study. Data sharing is not applicable to this article.
